# Commentary: Nix restores mitophagy and mitochondrial function to protect against PINK1/Parkin-related Parkinson's disease

**DOI:** 10.3389/fnmol.2017.00297

**Published:** 2017-09-19

**Authors:** Jin-Sung Park, Brianada Koentjoro, Carolyn M. Sue

**Affiliations:** ^1^Department of Neurogenetics, Kolling Institute, Northern Sydney Local Health District St. Leonards, NSW, Australia; ^2^Sydney Medical School—Northern, University of Sydney St. Leonards, NSW, Australia

**Keywords:** Parkinson's disease, Parkin, PINK1, mitophagy, nix

Parkinson's disease (PD) is a debilitating neurodegenerative disease affecting more than 1% of the population aged >65 years (Mehta et al., 2007). PD is caused by progressive loss of substantia nigra dopaminergic neurons (SN-DA) and the motor symptoms of PD such as tremor, bradykinesia and rigidity typically begin to manifest when about 60% of SN-DA are degenerated (Dauer and Przedborski, 2003). Although the mechanism underlying PD-linked neurodegeneration remains elusive, growing evidence suggests mitochondrial dysfunction in the pathogenesis of sporadic and familial PD (Schapira, 2008). Consistently, several genes which have been associated with PD are known to cause mitochondrial dysfunction.

Among the PD-linked genes, loss-of-function mutations in *PINK1* (PARK6; MIM# 608309) and *Parkin* (PARK2; MIM# 602544) are the most frequent cause of autosomal recessive early-onset PD (Klein and Westenberger, 2012). Functionally, PINK1 and Parkin play a crucial role in maintaining healthy mitochondria by regulating biogenesis, morphology, trafficking, and degradation (Scarffe et al., 2014), and recent advances greatly increased our understanding on their role in the autophagy-dependent selective degradation of dysfunctional mitochondria, termed mitophagy (reviewed in Youle and Narendra, 2011; Nguyen et al., 2016; Figure 1); Depolarization of mitochondria due to damage or aging stabilizes PINK1 on the outer mitochondrial membrane (OMM), which subsequently recruits Parkin to the dysfunctional mitochondria (Narendra et al., 2008). Then, Parkin, activated by PINK1-mediated phosphorylation (Narendra et al., 2010; Shiba-Fukushima et al., 2012), facilitates mitophagy by ubiquitinating OMM proteins to which the autophagosome receptor, microtubule-associated protein 1 light chain 3 (LC3), binds with help of polyubiquitin-binding adaptors (Youle and Narendra, 2011). Therefore, it is widely accepted, although *in vivo* evidence is still lacking due to the technical inability to monitor this process, that loss of PINK1 or Parkin induces impairment in mitophagy and accumulation of dysfunctional mitochondria, leading to nigrostriatal neurodegeneration, and PD (Scarffe et al., 2014). Intriguingly, several studies reported Parkin inactivation by structural modification such as S-nitrosylation (Chung et al., 2004), phosphorylation (Ko et al., 2010), and dopamine (LaVoie et al., 2005) in sporadic PD brains, suggesting the broader involvement of Parkin and impaired mitophagy in the pathogenesis of PD. In addition to the Parkin/PINK1-mediated pathway, several molecules including Fun14 domain-containing protein 1 (FUNDC1; Liu et al., 2012), FK506 binding protein 8 (FKBP8; Bhujabal et al., 2017), PINK1 (Lazarou et al., 2015), Autophagy and beclin 1 regulator 1 (AMBRA1; Strappazzon et al., 2015), and Gp78 (Fu et al., 2013) have been found to mediate mitophagy in a Parkin-independent manner.

**Figure 1:**
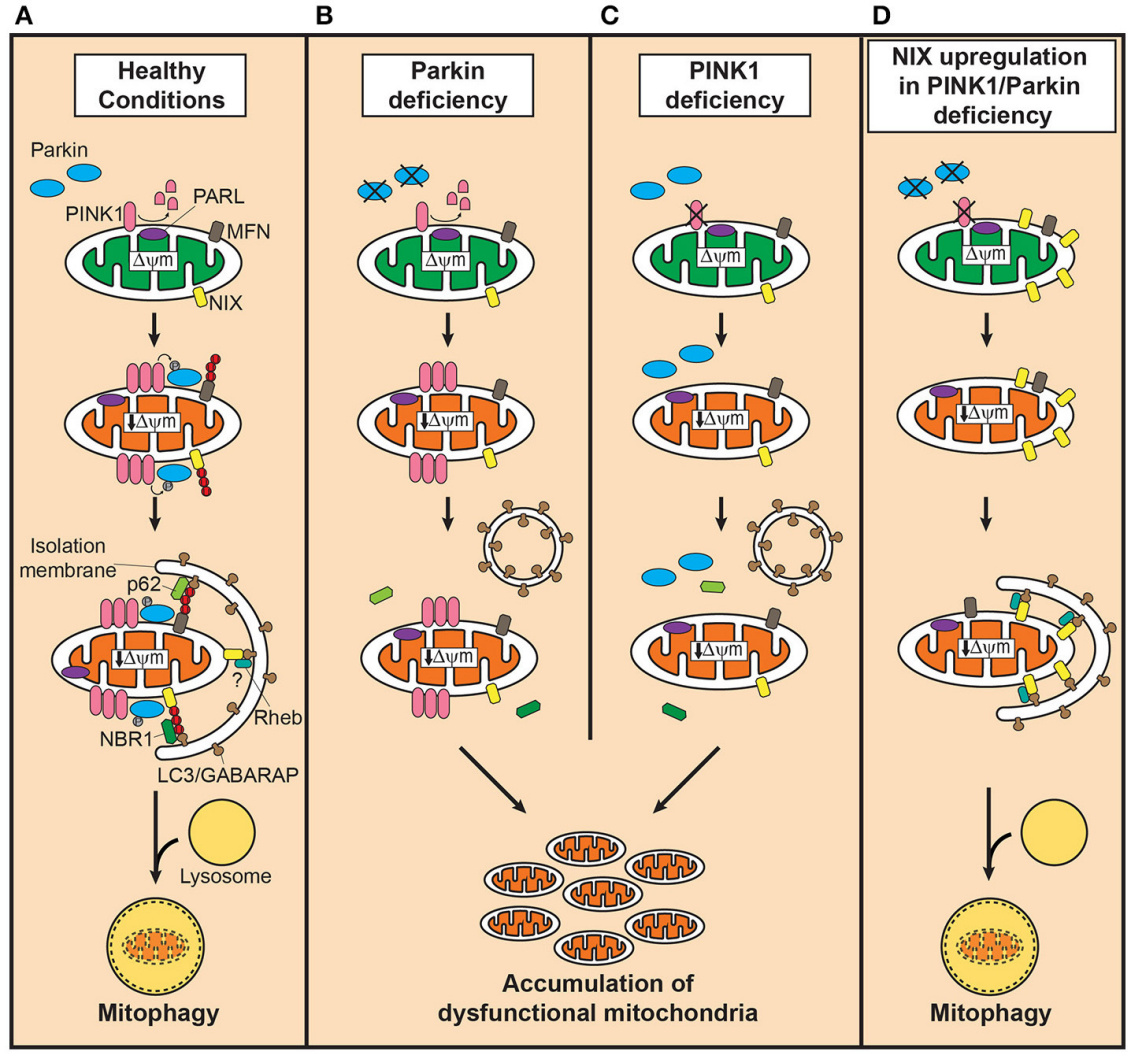
Defective mitophagy in PINK1/Parkin-related Parkinson's disease and the compensatory role of Nix in restoring mitophagy. **(A)** In healthy mitochondria, PINK1 is cleaved by presenilin-associated rhomboid-like protease (PARL). Dissipation of mitochondrial membrane potential decreases due to damage or aging, stabilizes PINK1 on the outer mitochondrial membrane (OMM) which in turn recruits Parkin to dysfunctional mitochondria. Parkin, activated by PINK1-mediated phosphorylation, ubiquitinates OMM such as mitofusins (MFN) to which the autophagosomal receptor microtubule-associated protein 1 light chain 3 (LC3)/gamma-aminobutyric acid receptor-associated protein (GABARAP) binds with polyubiquitine-binding proteins such as p62, optineurin and nuclear dot protein 52 kDa, leading to engulfment of dysfunctional mitochondria into autophagosomes. In the presence of Parkin, NIP3-like protein X (Nix) facilitates mitophagy by Parkin-mediated ubiquitination and recruitment of the polyubiquitine-binding protein neighbor of BRCA1 (NBR1). Although, Nix may mediate mitophagy independently of Parkin, its contribution is likely minor. Degradation of engulfed mitochondria occurs by fusion of autophagosomes with lysosomes. **(B)** In Parkin deficiency, accumulation of PINK1 occurs on the OMM of dysfunctional mitochondria, but the downstream events of polyubiquitination and subsequent recruitment of adaptor proteins fail to take place. **(C)** PINK1 deficiency, on the contrary, leads to failure in recognizing dysfunctional mitochondria, severely curtailing the efficiency of mitophagy. In both conditions, dysfunctional mitochondria accumulate due to impairment in inducing mitophagy. **(D)** Upregulation of Nix in PINK1 or Parkin deficiency, increases presence of Nix in dysfunctional mitochondria and mediates mitophagy by forming a complex with LC3/GABARAP and ras homolog enriched in brain protein (Rheb), maintaining mitochondrial quality control system and function.

The penetrance rate of Parkin-mediated PD is almost 100% (Schulte and Gasser, 2011), but recently we identified an asymptomatic homozygous *Parkin* mutation carrier (MC) who, despite the complete loss of Parkin, has not developed PD in her seventies (Koentjoro et al., 2012), suggesting the existence of a protective mechanism against Parkin deficiency. MC-derived fibroblasts showed sound mitochondrial function as shown by normal mitochondrial membrane potential and ATP production with well-preserved mitochondrial respiration, and intact mitophagy, indicating the presence of a Parkin-independent mitophagy (Koentjoro et al., 2017). As the molecular mechanism, the mitochondrial autophagy receptor Nip3-like protein X [Nix; also known as BCL2/adenovirus E1B 19 kDa interacting protein 3-like (BNIP3L)] was found to be responsible for maintaining mitochondrial quality by mediating mitophagy.

Nix, originally identified as a proapoptotic protein, has been known in association with mitophagy occurring in the development of reticulocytes (Ney, 2015). Although, a large part of the mechanism underlying Nix-mediate mitophagy such as recognition of dysfunctional mitochondria remains unknown, Nix mediates the isolation of mitochondria into autophagosomes through forming a protein complex with LC3/gamma-aminobutyric acid receptor-associated protein (GABARAP; Novak et al., 2010; Figure 1). Ras homolog enriched in brain protein (Rheb) has been shown to facilitate this process by promoting the interaction between Nix and LC3/GABARAP (Melser et al., 2013). In the presence of PINK1/Parkin-mediated mitophagy, Nix seems to function downstream of Parkin as a substrate; Nix, ubiquitinated by Parkin, binds to LC3/GABARAP through interaction with neighbor of BRCA1 (NBR1; Gao et al., 2015).

Contrary to the causative role of impaired mitophagy in PINK1/Parkin-related PD, the observation in MC on the ability of Nix to independently mediate mitophagy and thereby maintain normal mitochondrial function suggests that increased levels of Nix may compensate for the loss of PINK1/Parkin-mediated mitophagy in PD. Indeed, overexpression of Nix restored the cellular ability to activate mitophagy without triggering an aberrant increase in mitochondrial degradation or apoptosis, and improved mitochondrial energy production in cell lines derived from PINK1/Parkin-related PD patients (Koentjoro et al., 2017). Furthermore, induction of Nix expression using phorbol 12-myristate 13-acetate elicited a similar effect of restoring mitophagy, demonstrating the usefulness of Nix as a therapeutic target in drug development and human application. Taken together, these findings strongly support that Nix is responsible for prevention of PD as well as PD-associated neurodegeneration in MC, and has a therapeutic potential for PINK1/Parkin-related PD as a neuroprotective treatment.

Although, our study suggests Nix-mediated pathway as an innovative avenue to treat PD, there are key questions to be answered for clinical application of Nix. Particularly, the protective effect of Nix in animal models and the mechanisms underlying Nix induction and Nix-mediated mitophagy need to be urgently clarified. Also, knowledge on the expression of Nix in SN-DA of PD patients and animal models is of great interest. In addition, further evidence on the involvement of mitophagy in PD pathogenesis, especially sporadic cases, would also be beneficial.

Mitochondrial dysfunction has been shown to underlie neurodegeneration associated with PD. As the cause of mitochondrial dysfunction, defective mitophagy by loss of PINK1/Parkin has been shown in early-onset familial PD while evidence is growing in sporadic PD, broadening applicability of our approach to restore mitophagy as a treatment for PD. In this context, the newly identified compensatory role of Nix in mediating PINK1/Parkin-independent mitophagy provides a promising therapeutic target to treat mitochondrial dysfunction, which may lead to the development of a neuroprotective therapy for PD.

## Author contributions

All authors listed, have made substantial, direct, and intellectual contribution to the work, and approved it for publication.

### Conflict of interest statement

The authors declare that the research was conducted in the absence of any commercial or financial relationships that could be construed as a potential conflict of interest.
